# A history of second hand smoke exposure: are we asking the right questions?

**DOI:** 10.3389/fphys.2013.00025

**Published:** 2013-02-20

**Authors:** Mardi A. Crane-Godreau, Peter Payne

**Affiliations:** Department of Microbiology and Immunology, Geisel School of Medicine at DartmouthLebanon, NH, USA

This commentary is written to accompany a special research topic, *Second Hand Smoke and COPD: lessons from animal studies*, hosted by *Frontiers in Respiratory Physiology.*

Model systems allow researchers to study disease, to tease out cause and effect and mechanisms of action, and to conduct the preliminary studies of the safety and efficacy of treatments. The underlying objective of the research is, of course, to improve healthcare outcomes. Pointing out the difficulty of studying a disease that takes decades to develop in humans, this collection of articles focuses on what has been learned from studies using models of COPD. It also presents an opportunity to circle back to the implications of the research and to ask a most basic question: do healthcare providers recognize the impact of second hand smoke (SHS) exposure on health and, if they do, are they asking the right questions?

The 2006 Surgeon General's Report, “Health Consequences of Involuntary Exposure to Tobacco Smoke” (Surgeon General, 2006) documents the health implications of exposure to SHS, including firm evidence that SHS contributes to coronary and lung disease, lung cancer, premature death in adults, slow lung development, SIDS, asthma, and ear infections in children, as well as suggestive evidence that implicate SHS in COPD, asthma, breast cancer, and nasal sinus cancer in adults, and leukemia, lymphoma, and brain tumors in children. The report indicates that there is no risk-free level of SHS.

Despite evidence that SHS is a risk factor for disease, most healthcare organizations and many physicians fail to ask patients about their history of SHS exposure. The implications of that failure are considerable because knowledge of a patient's history of SHS exposure enables providers to make better-informed decisions about what to include in each patient's examination and lab tests, and how to conduct long-term monitoring, as well as alerting the patient to the need for measures to help them avoid further smoke exposure.

An example of a medical history of SHS exposure on health status can be seen in the development of lung disease in flight attendants, a group who historically worked in SHS filled aircraft. Recent research has revealed decreased exercise tolerance, decreased diffusing capacity with decreased pulmonary capillary recruitment as well as air trapping and airway obstruction in never-smoking flight attendants who were exposed prior to the ban on smoking in aircraft (Arjomandi et al., 2009, 2012). US aircraft became smoke-free by 1990 for flights less than 6 h, and by 2000 all US carriers were required to be smoke-free. These flight attendants, despite never smoking, have evidence of lung disease 10–20 years after their SHS exposure. For patients with a history of SHS exposure, failure of providers to include the right questions may leave risks hidden and opportunities for early intervention lost.

The American Academy of Pediatrics (AAP) has pioneered efforts to encourage physicians to ask the right questions regarding the exposure of children to SHS. Recognizing that SHS is a major contributor to childhood morbidity and mortality, the AAP has made asking about SHS exposure an essential part of regular pediatric care. This practice of asking the right questions could serve as a model for healthcare providers worldwide.

Prevention of disease progression due to SHS of course has clear implications for patients' suffering and their ability to continue to function in society. SHS exposure also has significant financial implications for healthcare payers. As the health care system in the US moves from a fee-for-service to a fee-for-results structure, prevention of disease and promotion of wellness become much more important. Early recognition of the presence of risk factors for the development of tobacco smoke-related disease enables intervention before more severe conditions arise; from the most pragmatic perspective, advanced disease is simply more costly to treat.

The importance of asking about SHS exposure is not limited to clinical care. Researchers too can improve study design and outcomes when they recognize the impact of SHS exposure. In human studies that look for risk factors for disease, the failure to ask about an individual's history of SHS exposure may lead to confusing or less significant results. For instance, when looking for the impact of smoking on cancer incidence, osteoporosis, or lung disease, placing those who have been exposed to SHS in the category “non-smoker,” as many studies have done, simply makes no sense.

Additional reason for asking about a history of SHS exposure is suggested by the links between Vitamin D deficiency and the development of COPD. Our own laboratory has recently focused on the impact of Vitamin D deficiency on the development and progression of SHS-related emphysema in a mouse model. Epidemiologic studies by Janssens and colleagues demonstrated low serum levels of 25-hydroxyvitamin D in advanced stage COPD patients, as well as an increased incidence of COPD in individuals who carry a mutation in one or more of the genes involved in vitamin D metabolism (Janssens et al., 2009a, 2011). Our model of cigarette smoke exposure with vitamin D deficiency supports the hypothesis that low levels of 25-hydroxyvitamin D may contribute to the severity of SHS-related emphysema. Given the multiple roles of vitamin D in infection, cardiovascular disease, and osteoporosis (all comorbidities of COPD), it seems prudent for health care providers to screen for vitamin D levels in at-risk patients (Holick, 2004; Lee et al., 2008; Janssens et al., 2009b). Without knowing a patient's history of SHS exposure, vitamin D screening might not be considered.

COPD is the third leading cause of death worldwide. While lung damage from COPD is irreversible, recognition of the early stages of the disease allows providers to intervene so as to slow or arrest disease progression. While smoking is widely acknowledged as a risk factor, the danger of SHS exposure is significantly under-recognized. Since the initial symptoms of COPD are hard to detect, increased awareness of SHS exposure as a risk factor should alert caregivers to screen for early signs of disease. This would give them an earlier indicator of increased disease risk and provide crucial additional time in which to mitigate the onset and progression of disease. Counseling to avoid smoke exposure, monitoring for early evidence of changes in lung function, vitamin D monitoring, and early inclusion of interventions such as exercise, respiratory training, and mind-body practices such as Qigong or Tai Chi, can all be part of a strategy to control the disease process.

There is an ongoing emphasis on improving outcomes and lowering costs of health care. The AAP has taken the lead in encouraging a simple and inexpensive approach to lowering the impact of SHS exposure: simply talking to patients about it and taking it into account when considering the patient's overall health. It's a winning strategy; asking about the history of SHS exposure as part of the course of regular care would give providers greater opportunities for early intervention and patients opportunities for improved long-term health.
